# 4.2 A TECHNOLOGY-ENHANCED INTERVENTION TO REDUCE THE DURATION OF UNTREATED PSYCHOSIS THROUGH RAPID IDENTIFICATION & ENGAGEMENT

**DOI:** 10.1093/schbul/sby014.009

**Published:** 2018-04-01

**Authors:** Tara Niendam, Rachel Loewy, Mark Savill, Monet Meyer, Adi Rosenthal, Kevin Delucchi, Tyler Lesh, Haley Skymba, J Daniel Ragland, Howard Goldman, Rosemary Cress, Richard Kravitz, Cameron Carter

**Affiliations:** 1 University of California, Davis School of Medicine; 2 Weill Institute for Neurosciences, University of California, San Francisco; 3 University of Maryland School of Medicine

## Abstract

**Background:**

Reducing the duration of untreated psychosis (DUP) is essential to improve long-term outcome in young people with first episode of psychosis (FEP). The US “standard of FEP care” focuses on targeted provider education regarding FEP signs and symptoms to motivate referrals to FEP coordinated specialty care (CSC) services. However, a recent US multisite CSC trial showed a median DUP of 74.5 weeks, suggesting the current approach to engage referral sources is not sufficient to reduce DUP to proposed international standards of 12 weeks. This cluster-randomized controlled trial assesses whether standard targeted provider education plus novel technology-enhanced screening using the Prodromal Questionnaire-Brief version (PQ-B) identifies more individuals with FEP, earlier in their illness, compared to standard targeted provider education alone.

**Methods:**

Twenty-two sites were randomized within 3 strata [community mental health, CMH (N=10), middle/high schools, SCH (N=8), primary care, PC (N=4)] to 1 of 2 intervention arms [Education alone (TAU) vs Education + Electronic Screening (Active)]. Active sites screened eligible individuals ages 12–30 at initial presentation for mental health concerns and referred those who passed a liberal PQ-B cut off score for phone evaluation by the CSC clinic. TAU sites referred individuals for phone evaluation based on clinician judgment. Phone evaluations assessed eligibility for FEP services and DUP. Preliminary analyses examined the number of FEP referrals and length of DUP in each arm.

**Results:**

Active sites effectively implemented electronic screening within their settings. Of the 822 individuals electronically screened at Active sites between June 2015 and July 2017, 43.2% scored above the PQ-B cutoff (mean±SD PQ-B score=21.25 ± 20.75; median=15; range = 0–95; IQR = 3–35). One in 8 individuals who completed the tablet were identified as experiencing threshold psychosis. Across both Active and TAU sites, 511 individuals were identified, 422 individuals agreed to be referred, and 319 completed a phone interview to determine eligibility: 33.23% reported attenuated and 36.68% fully psychotic symptoms. Active sites identified significantly more individuals with threshold psychosis (p<.001) than TAU. No difference in median days of DUP was observed across arms.

**Discussion:**

Preliminary results show the feasibility of electronic screening across various community settings and showed a 3.5 times higher identification rate for electronic screening of self-reported psychosis spectrum symptoms than clinician-based identification alone. Reasons for the lack of difference in DUP will be discussed. While the screening method may shorten the time from entry into mental health care and referral to specialty care treatment, significant DUP reduction may require interventions to reduce time to the first mental health contact. The next phase of the project will examine impact of clinic-based versus community-based treatment engagement to reduce barriers to initiating CSC care.

